# A mouse SWATH-mass spectrometry reference spectral library enables deconvolution of species-specific proteomic alterations in human tumour xenografts

**DOI:** 10.1242/dmm.044586

**Published:** 2020-07-14

**Authors:** Lukas Krasny, Philip Bland, Jessica Burns, Nadia Carvalho Lima, Peter T. Harrison, Laura Pacini, Mark L. Elms, Jian Ning, Victor Garcia Martinez, Yi-Ru Yu, Sophie E. Acton, Ping-Chih Ho, Fernando Calvo, Amanda Swain, Beatrice A. Howard, Rachael C. Natrajan, Paul H. Huang

**Affiliations:** 1 Division of Molecular Pathology, The Institute of Cancer Research, London SW3 6JB, UK; 2 The Breast Cancer Now Toby Robins Research Centre, The Institute of Cancer Research, London SW3 6JB, UK; 3 Tumour Profiling Unit, The Institute of Cancer Research, London SW3 6JB, UK; 4 Stromal Immunology Group, MRC Laboratory for Molecular Cell Biology, University College London WC1E 6BT, London, UK; 5 Department of Oncology, University of Lausanne, Lausanne CH-1066, Switzerland; 6 Ludwig Institute for Cancer Research, Lausanne CH-1066, Switzerland; 7 The Tumour Microenvironment Team, Institute of Biomedicine and Biotechnology of Cantabria, Santander 39011, Spain

**Keywords:** Mass spectrometry, SWATH-MS, Proteomics, Mouse, Xenografts, DCIS, Breast cancer

## Abstract

SWATH-mass spectrometry (MS) enables accurate and reproducible proteomic profiling in multiple model organisms including the mouse. Here, we present a comprehensive mouse reference spectral library (MouseRefSWATH) that permits quantification of up to 10,597 proteins (62.2% of the mouse proteome) by SWATH-MS. We exploit MouseRefSWATH to develop an analytical pipeline for species-specific deconvolution of proteomic alterations in human tumour xenografts (XenoSWATH). This method overcomes the challenge of high sequence similarity between mouse and human proteins, facilitating the study of host microenvironment-tumour interactions from ‘bulk tumour’ measurements. We apply the XenoSWATH pipeline to characterize an intraductal xenograft model of breast ductal carcinoma *in situ* and uncover complex regulation consistent with stromal reprogramming, where the modulation of cell migration pathways is not restricted to tumour cells but also operates in the mouse stroma upon progression to invasive disease. MouseRefSWATH and XenoSWATH open new opportunities for in-depth and reproducible proteomic assessment to address wide-ranging biological questions involving this important model organism.

## Abstract (precis)

**Editor's choice:** This paper presents the MouseRefSWATH mouse reference spectral library as a standardized community resource for SWATH-mass spectrometry studies and the XenoSWATH pipeline for species-specific deconvolution of human xenograft proteomic data.

## INTRODUCTION

Mass spectrometry (MS) has become an essential tool for contemporary proteomic research in life sciences. Conventional data-dependent acquisition (DDA) mode, where a fixed number of the most abundant precursor ions in survey scans is automatically selected for fragmentation, enables the identification of thousands of proteins in a single MS experiment. However, the stochastic nature of peptide precursor ion selection in DDA leads to low reproducibility in peptide identification between experimental runs ranging from 35% to 60% (Tabb et al., 2010; Krasny et al., 2018; Bruderer et al., 2015; Barkovits et al., 2020). Sequential window acquisition of all theoretical mass spectra (SWATH-MS) or data-independent acquisition mass spectrometry (DIA-MS) is a next-generation label-free quantification method that enables highly reproducible peptide identification (ranging from 80% to 98%) and more accurate quantification in large-scale proteomic analyses across multiple experiments (Gillet et al., 2012; Muntel et al., 2019; Collins et al., 2017; Bruderer et al., 2015; Barkovits et al., 2020). In contrast to DDA, the SWATH methodology captures all peptide precursor ions in a sample under investigation and fragments these ions in a series of wide and adjacent isolation windows that cover the entire mass to charge ratio (m/z) range measured (Gillet et al., 2012). This approach leads to the generation of a final digital proteome map comprising fragmentation spectra of all detected peptides in each sample for subsequent *in silico* analysis (Guo et al., 2015). The ability to capture all peptide precursor ions dramatically improves reproducibility in protein identification across multiple samples. These quantitative digital proteome maps have been shown to have wide applications in medical research, such as the discovery of potential biomarkers and therapeutic targets (Cecchettini et al., 2019; Gao et al., 2017; Miyauchi et al., 2018; Hou et al., 2016; Xu et al., 2018), as well as tumour proteotyping for cancer stratification and classification (Bouchal et al., 2019; Zhu et al., 2019).

Key to the success of SWATH-MS is the use of retention time-calibrated spectral libraries to identify and quantify peptides from the complex fragment ion mass spectra encoded within digital proteome maps (Bruderer et al., 2016). Although most published SWATH-MS studies have utilized study-specific experimentally derived spectral libraries, this approach is time consuming, and is prone to wide variation between laboratories due to the lack of standardization in DDA data acquisition and library generation. Such variation results in study-specific bias and poor inter-laboratory reproducibility. The recent development of the algorithms that control for false discovery rate (FDR) in SWATH-MS datasets has opened up the possibility of building large reference spectral libraries that can be readily shared by the community, increasing data reproducibility across laboratories and accelerating SWATH-MS experiments without the need to generate study-specific spectral libraries (Reiter et al., 2009; Rosenberger et al., 2014).

Several such large reference spectral libraries have been built and deposited into repositories such as SWATHAtlas (www.SWATHatlas.org), including libraries for human (Rosenberger et al., 2014), fruit fly (Fabre et al., 2017), zebrafish (Blattmann et al., 2019) and yeast (Picotti et al., 2013). There is, however, currently no publicly available comprehensive mouse reference spectral library. The mouse as a model organism has and continues to be used extensively in developmental biology and medical research due to the complex genetics and physiological systems that mammals share. The array of innovative genetic strategies available for engineering mice has revolutionized our understanding of multiple human diseases, including cancer, diabetes, autoimmune disease and heart disease, amongst others. Furthermore, immunocompromised mouse models have been used for more than a decade as hosts for human tumour (both cell line and patient-derived) xenografts, which have served as an essential tool to investigate the factors that drive carcinogenesis as well as to evaluate cancer therapeutics (Richmond and Su, 2008). Developing a comprehensive mouse reference spectral library will facilitate the application of SWATH-MS to address key research questions involving this widely used model organism. A number of study-specific mouse spectral libraries have previously been reported (Caron et al., 2015; Williams et al., 2018; von Ziegler et al., 2018; Malmstrom et al., 2016); for instance, a spectral library that is comprised of the mouse immunopeptidome, containing 1573 peptides presented by MHC class I molecules (Caron et al., 2015), and a spectral library reported by Williams et al. (2018), which contains 5152 proteins (30% of mouse proteome) generated from five organs. Notably, in the aforementioned human tumour xenograft models, a small number of DDA-MS studies have described the deconvolution of host (mouse) versus tumour (human) proteomic alterations from ‘bulk tumour’ proteomes (Rajcevic et al., 2009; Wang et al., 2017; Wildburger et al., 2015), but these studies lack the reproducibility afforded by SWATH-MS, which has limited our ability to robustly study the role of the tumour microenvironment in driving tumour initiation and progression.

In this study, we present a comprehensive mouse reference spectral library (MouseRefSWATH) generated from 15 distinct mouse organs and cellular samples. MouseRefSWATH was built from 254 individual MS experiments and is composed of transitions for 167,138 proteotypic peptides from 10,597 proteins representing 62.2% of manually validated mouse protein-encoding genes (SwissProt database). The performance of MouseRefSWATH was evaluated in two publicly available SWATH-MS datasets, which showed both qualitative and quantitative reproducibility when compared to published study-specific spectral libraries. We further report the development of a novel application of MouseRefSWATH for SWATH-MS-based mapping of species-specific temporal proteomic alterations in an orthotopic tumour xenograft model of breast ductal carcinoma *in situ* (DCIS). Utilizing this approach, we reveal, for the first time, simultaneous temporal regulation of cell migration pathways in both the host mouse mammary gland and human tumour cells during the course of DCIS to invasive breast cancer (IBC) progression. This XenoSWATH pipeline for species-specific deconvolution of ‘bulk tumour’ proteomics data provides a useful tool with broad applications for the analysis of host microenvironment-tumour interactions in xenograft models without the need for prior separation of host and tumour cell populations. The MS raw data and MouseRefSWATH spectral library for this study are available via ProteomeXchange with identifier PXD017209 and via SWATHatlas.org and PeptideAtlas.org with identifier PASS01569.

## RESULTS

### Proteomic analysis of murine tissue and cells

**Fig. 1.**
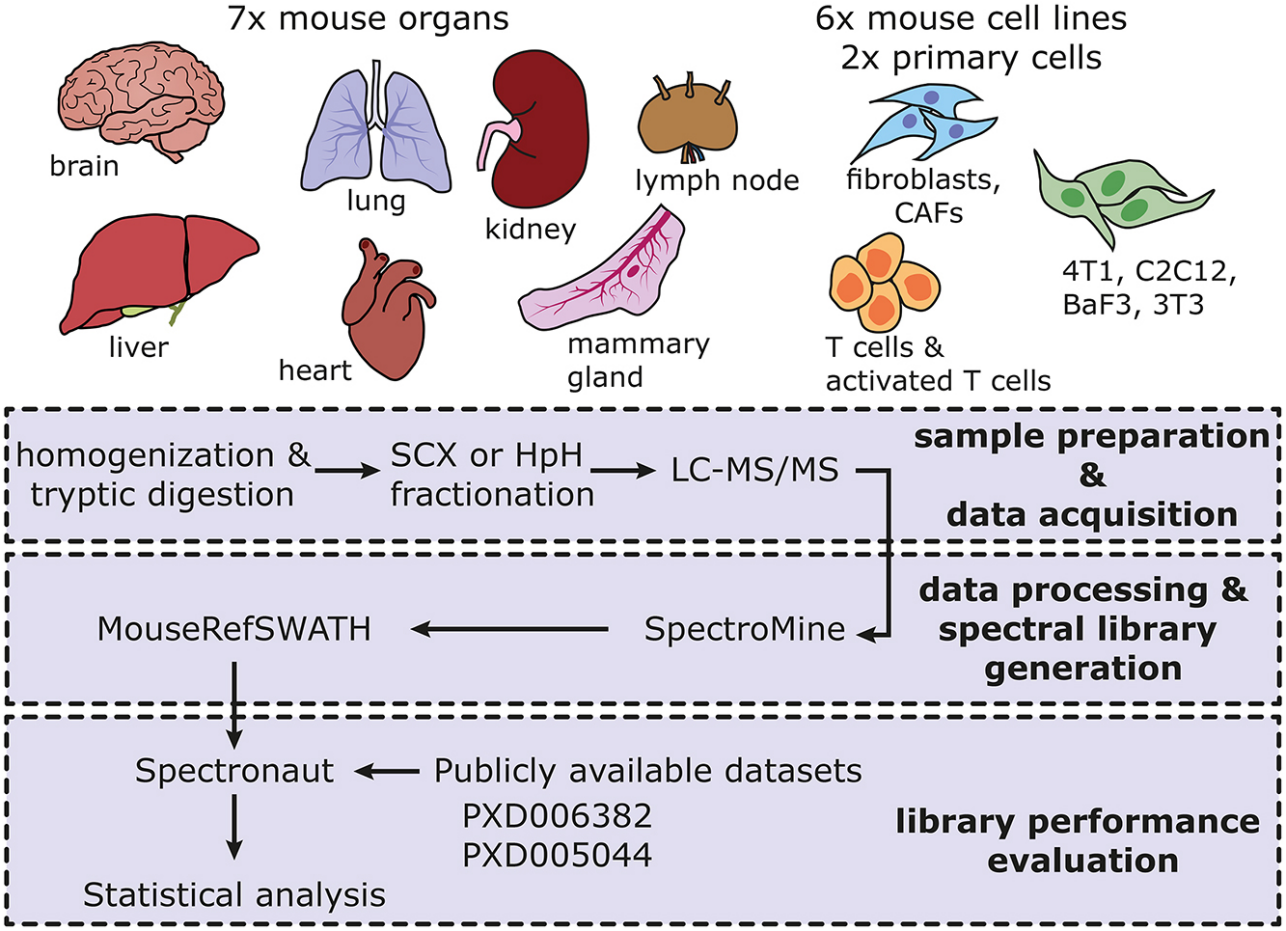
**Overview of the sample types and schematic workflow used to build the MouseRefSWATH reference spectral library.** Fifteen unique sample types comprising seven mouse organs, two primary cell types and six mouse cell lines were analysed to maximize proteome coverage. The workflow consists of a first step of sample preparation and data acquisition of each sample by data-dependent acquisition (DDA)-based liquid chromatography–tandem mass spectrometry (LC-MS/MS). In the second step, the resulting proteomic data were subjected to processing and spectral library generation using the combined Search Archives approach in the SpectroMine software. The subsequent evaluation of the performance of the MouseRefSWATH library was undertaken with the Spectronaut software utilizing two publicly available datasets. CAF, cancer-associated fibroblast; HpH, high pH; SCX, strong cation exchange.

To maximize coverage of the mouse proteome in the reference spectral library, we performed DDA proteomic analysis of a comprehensive range of seven murine organs: heart, brain, lung, liver, kidney, lymph node and mammary gland (Fig. 1 and Table 1). In addition, we undertook proteomic profiling from primary CD8^+^ T-lymphocytes (non-stimulated and activated) as well as immortalized murine cell lines including normal (NF1) and cancer-associated (CAF1) fibroblasts (Calvo et al., 2013) and commercially available cell lines NIH-3T3, C2C12, 4T1 and Ba/F3 of diverse tissue origin (Table 1). As outlined in the workflow shown in Fig. 1, extracted proteins from each sample type were digested by trypsin and the resulting peptide mixture was subjected to offline fractionation in the first dimension either by strong cation exchange (SCX) chromatography or reverse-phase chromatography in high pH (HpH-RP) (Table 2). To calibrate the chromatographic retention time for individual DDA runs, prior to liquid chromatography–tandem mass spectrometry (LC-MS/MS) analysis, each fraction was spiked with the indexed retention time (iRT) calibration standard that contains a mixture of 11 synthetic peptides (Escher et al., 2012; Bruderer et al., 2016). Peptides from each fraction were subsequently analysed by LC-MS/MS in DDA mode and the acquired data were processed by SpectroMine (Keller et al., 2005; Deutsch et al., 2010). An average of 3200-6600 proteins were identified across the different samples as shown in Table 2.

**Table 1.**
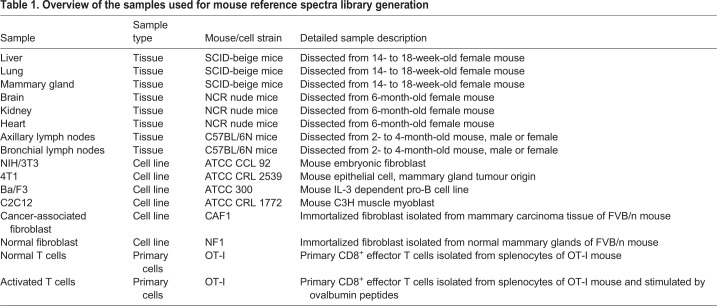
**Overview of the samples used for mouse reference spectra library generation**

**Table 2.**
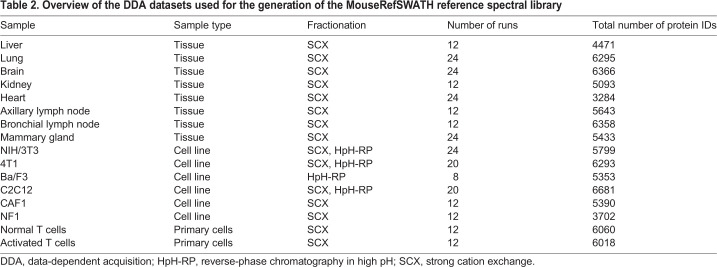
**Overview of the DDA datasets used for the generation of the MouseRefSWATH reference spectral library**

### Building the MouseRefSWATH spectral library

**Fig. 2.**
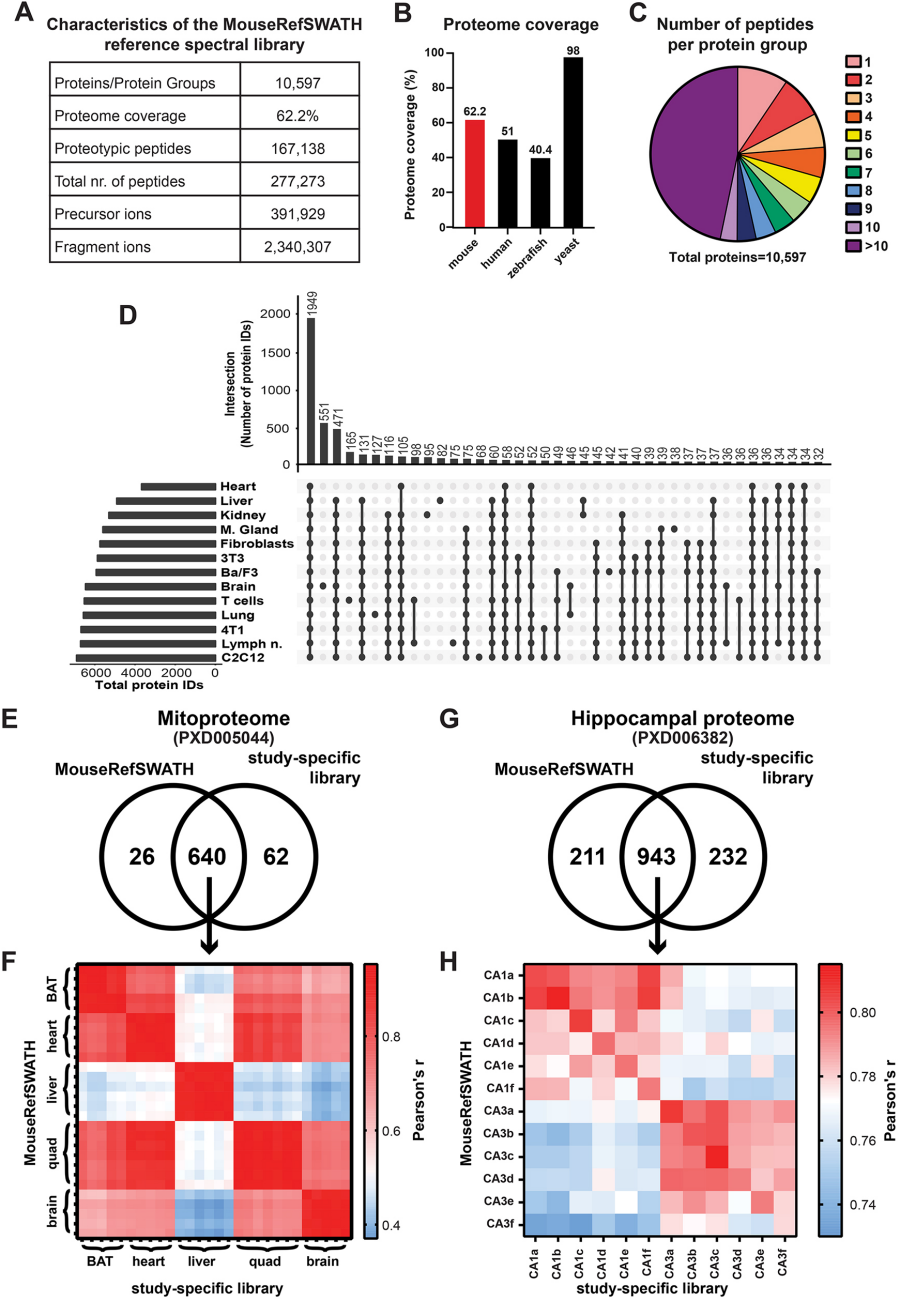
**Characteristics and performance of the MouseRefSWATH reference spectral library.** (A) Detailed characteristics of the generated MouseRefSWATH library. (B) Percentage proteome coverage of MouseRefSWATH library in comparison with published spectral libraries for other eukaryotic organisms, including human (Rosenberger et al., 2014), zebrafish (Blattman et al., 2019) and yeast (Picotti et al., 2013). (C) Peptide coverage of the individual proteins in the MouseRefSWATH library. 90.6% of the proteins are represented by more than one unique peptide. (D) Contribution plot of the tissue- or cell-specific proteins to the overall composition of the MouseRefSWATH library. The bar chart at the top depicts the total number of proteins identified in the overlap of datasets contributed by different organs/cell types as indicated in the dot plot below. The bar chart on the left indicates the total number of proteins identified in the individual datasets from each organ or cell type. (E) Venn diagram depicting overlap of mitochondrial proteins detected by the MouseRefSWATH library and study-specific library based on analysis of the PXD005044 dataset (Williams et al., 2018). (F) Similarity matrix showing Pearson's correlation coefficient of the 640 overlapping mitochondrial proteins, which were quantified by either the MouseRefSWATH library or the study-specific library. Five to eight animals were used for each tissue site. BAT, brown adipocyte tissue; quad, quadriceps. (G) Venn diagram depicting overlap of hippocampal proteins detected by the MouseRefSWATH library and study-specific library based on analysis of the PXD006382 dataset (von Ziegler et al., 2018). (H) Similarity matrix showing Pearson's correlation coefficient of the 943 overlapping hippocampal proteins, which were quantified by either the MouseRefSWATH library or the study-specific library. Two hippocampal areas (CA1 and CA3) from six animals (a-f) were used.

We built the mouse reference spectral library (MouseRefSWATH) from the datasets listed in Table 2 by employing the SpectroMine software (Fig. 1). Only unique protein-specific (proteotypic) peptides were used to generate the library, which contains 10,597 proteins (Fig. 2A), representing 62.2% coverage of manually annotated mouse protein-coding genes (SwissProt, 26/10/2018). Comparative analysis demonstrates a superior proteome coverage versus published spectral libraries of other higher eukaryotic organisms [human – 51% (Rosenberger et al., 2014) and zebrafish – 40.4% (Blattmann et al., 2019)] (Fig. 2B). Fig. 2C shows the distribution of unique peptides per protein group with 90.6% of proteins represented by >1 unique peptide in the spectral library and 46.7% of proteins being represented by >10 unique peptides. The contribution plot (Fig. 2D) shows that 1949 proteins (18.4%) in the MouseRefSWATH library were detected in all analysed sample types used in the generation of the library. The brain contributed the highest number of proteins (551, 5.2%) to the MouseRefSWATH library, followed by T cells (165, 1.6%) and lung (127, 1.2%).

### Evaluating the performance of the MouseRefSWATH spectral library

To demonstrate the utility of the MouseRefSWATH library and benchmark its performance against study-specific libraries generated as part of published SWATH-MS studies, we applied the reference spectral library to two publicly available SWATH-MS datasets focused on mitochondrial (Williams et al., 2018) and hippocampal (von Ziegler et al., 2018) proteins (Fig. 1). These datasets were generated by quadrupole time-of-flight instruments and consist of MS runs that have included the iRT calibration standard. It should be noted that the use of the MouseRefSWATH spectral library for retrospective and prospective analysis requires spiking of the iRT calibration standard so that the appropriate retention time calibration can be performed. Each dataset was analysed using either the original study-specific library generated by the authors or the MouseRefSWATH library.

In a recent mitoproteome study undertaken by Williams et al. (2018), a study-specific library based on DDA analysis of both total tissue lysates and mitochondria-enriched samples was utilized. We sought to assess the extent to which the MouseRefSWATH library was able to both qualitatively and quantitatively recapitulate the data generated by the study-specific library in the published study. The SWATH-MS data of mitochondrial proteins was downloaded from ProteomeXchange (PXD005044) and analysed using both libraries (Fig. 2E,F). The Williams et al. (2018) experimental dataset was generated from mitochondria enriched from five distinct murine tissues [brown adipose tissue (BAT), heart, liver, quadriceps, brain] from five to eight animals. Qualitatively, the MouseRefSWATH library identified 91.1% (640 proteins) of the mitoproteome (Fig. 2E) found using the study-specific library, with an additional 26 proteins identified specifically by the MouseRefSWATH library. An assessment of the quantification of the 640 overlapping proteins showed a good correlation between both libraries with Pearson's correlation coefficient r values ranging between 0.92 and 0.95 (Fig. 2F).

We undertook a similar comparative analysis on the study by von Ziegler et al. (2018), where the basal mouse hippocampal proteome was analysed utilizing a study-specific spectral library built from DDA data generated from the mouse hippocampus. For this analysis, the SWATH-MS experimental data of hippocampal area CA1 and CA3 from six different animals were analysed (Fig. 2G,H). The MouseRefSWATH library identified 80.2% (943 proteins) of hippocampus proteome found using the study-specific spectral library (Fig. 2G). The MouseRefSWATH library further identified 211 more proteins compared to the hippocampus-specific spectral library. Good correlation in protein quantification between the MouseRefSWATH and study-specific library for the overlapping 943 proteins was observed, with Pearson's correlation coefficient r values ranging between 0.78 and 0.81 across the six mice in the experiment (Fig. 2H). Taken together, our analysis demonstrates that applying the MouseRefSWATH to SWATH-MS datasets is routinely able to identify >80% of proteins identified using study-specific libraries with comparable quantification, highlighting its broad utility as a general reference library applicable for use in multiple mouse SWATH-MS datasets without the need to generate study-specific libraries.

### Developing the XenoSWATH analysis pipeline for deconvolution of mouse and human proteins in tumour xenograft SWATH-MS data

Mouse xenograft studies are one of the cornerstones of modern cancer research, where human tumour cells are typically grafted into a mouse host either subcutaneously or orthotopically as a means to evaluate oncogene and tumour suppressor gene function or investigate the therapeutic effects of drugs (Rosenbluh et al., 2012; Rosato et al., 2018). In these models, there are complex interactions between the human tumour cells and the host microenvironment, which play important roles in driving cancer progression and therapy response (Jansen et al., 2005; Hu et al., 2008; Lyons et al., 2011). To study these interactions, previous proteomic studies have attempted to tackle this challenge by separating human tumour and murine host cells utilizing immunoaffinity cell-enrichment strategies prior to MS analysis (Kalita-de Croft et al., 2019). However, such methods are labour intensive, introduce sample preparation biases and disrupt the *in situ* architecture and cellular interactions important for driving tumour biology. There have also been a small number of DDA-MS studies that have used either label-free methods or isobaric labelling to identify species-specific signalling in xenograft models of glioma and breast cancer (Wildburger et al., 2015; Rajcevic et al., 2009; Wang et al., 2017); however, these methods suffer from low reproducibility in peptide identification between individual experimental runs (Bruderer et al., 2015; Barkovits et al., 2020).

**Fig. 3.**
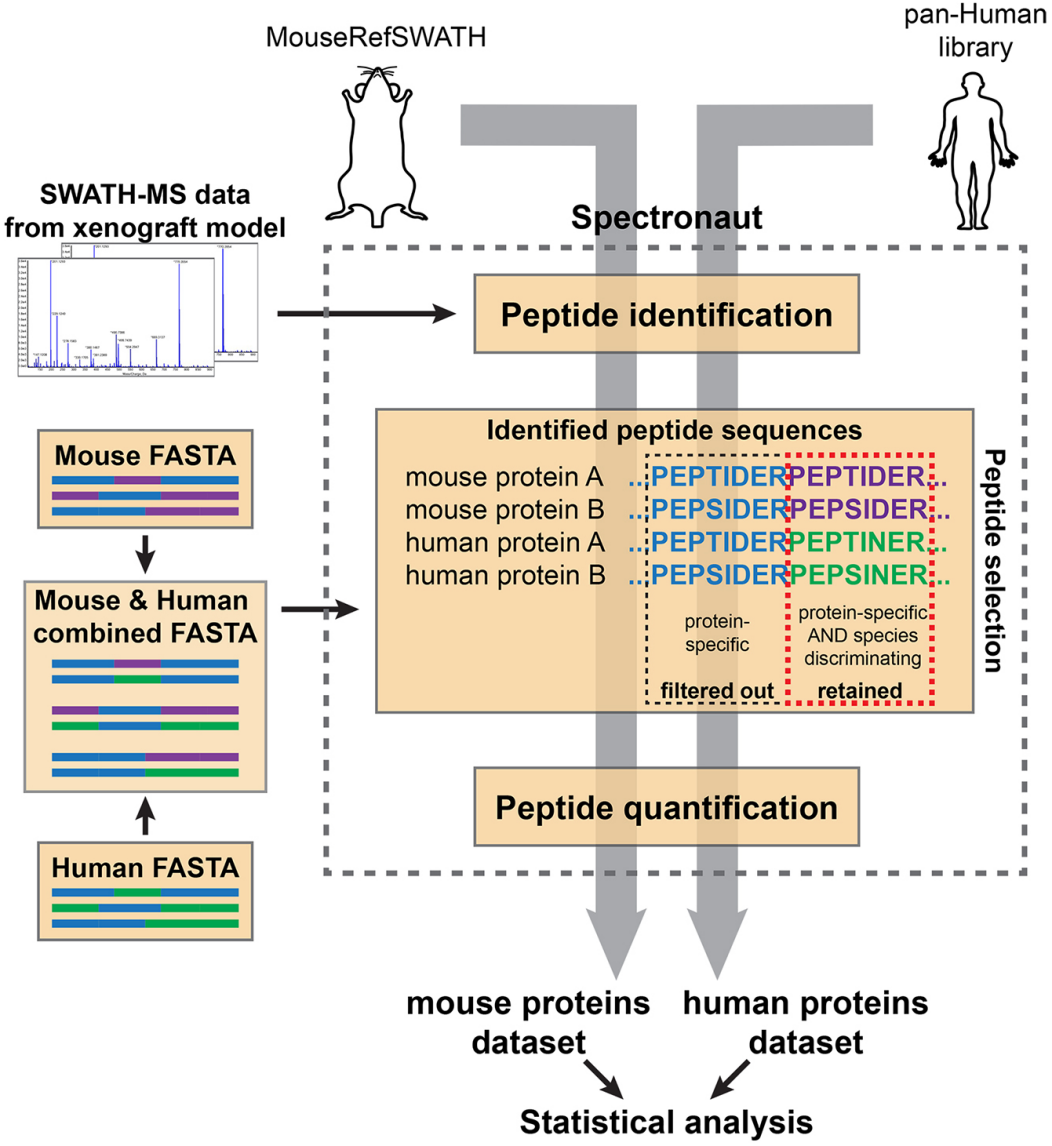
**XenoSWATH workflow of the species-specific deconvolution analysis pipeline.** The ‘bulk tumour xenograft’ acquired SWATH-MS data are first subjected to two separate searches by either the MouseRefSWATH or the pan-Human library in the Spectronaut software to identify protein-specific (proteotypic) peptides. A combined FASTA file of human and mouse *in silico* digested peptides is then generated to enable subsequent peptide quantification of species-discriminating proteotypic peptides. For peptide quantification, Spectronaut will compare the sequences of the identified peptides from either the MouseRefSWATH or pan-Human library searches with the peptides sequences in the combined FASTA file. Because protein-specific (proteotypic) peptides that are not species discriminating (blue) occur more than once in the combined FASTA file, Spectronaut filters these peptides out. Only peptides that are both protein specific and species discriminating (purple, mouse; green, human) in the combined FASTA file are retained and subjected to quantification. The output of this pipeline is two quantified datasets, one specific for mouse proteins and the other for human proteins.

To address these challenges, we developed a novel pipeline (XenoSWATH) to deconvolute species-specific proteomic profiles from SWATH-MS data obtained in mouse xenograft experiments. In this XenoSWATH workflow (Fig. 3), both the MouseRefSWATH library as well as a previously published pan-Human reference library (Rosenberger et al., 2014) were used in the Spectronaut software (Reiter et al., 2009). In the first step of data processing, peptides were identified by searching the acquired SWATH-MS data against either the MouseRefSWATH or pan-Human library. Both libraries contain proteotypic peptides, which can distinguish individual proteins; however, given that humans and mice share ∼70% of protein-coding sequences (Waterston et al., 2002), not all of these proteotypic peptides are also species discriminating. Therefore, to selectively quantify human and mouse proteins from ‘bulk tumour xenograft’ proteomic datasets, we focused on peptides that are both protein and species discriminating (see Fig. 3 for example). To achieve this, rather than using individual FASTA files comprising *in silico* digested peptides for either human or mouse proteins, we instead manually combined both files into a single FASTA file for peptide quantification. This modification to the data processing pipeline enables Spectronaut to compare sequences of the identified peptides from either the mouse or human reference spectral library with the sequences of the *in silico* digested peptides within the combined FASTA file, and filter out any peptides that are shared between human and mouse. This leads to the retention of only peptides that are both proteotypic and species discriminating for peptide quantification. The resultant output from this XenoSWATH pipeline is two datasets – one consisting entirely of murine proteins and the other of human proteins.

### SWATH-MS analysis and deconvolution of tumour (human) and host mammary gland (mouse) proteomic alterations in a xenograft model of DCIS progression

**Fig. 4.**
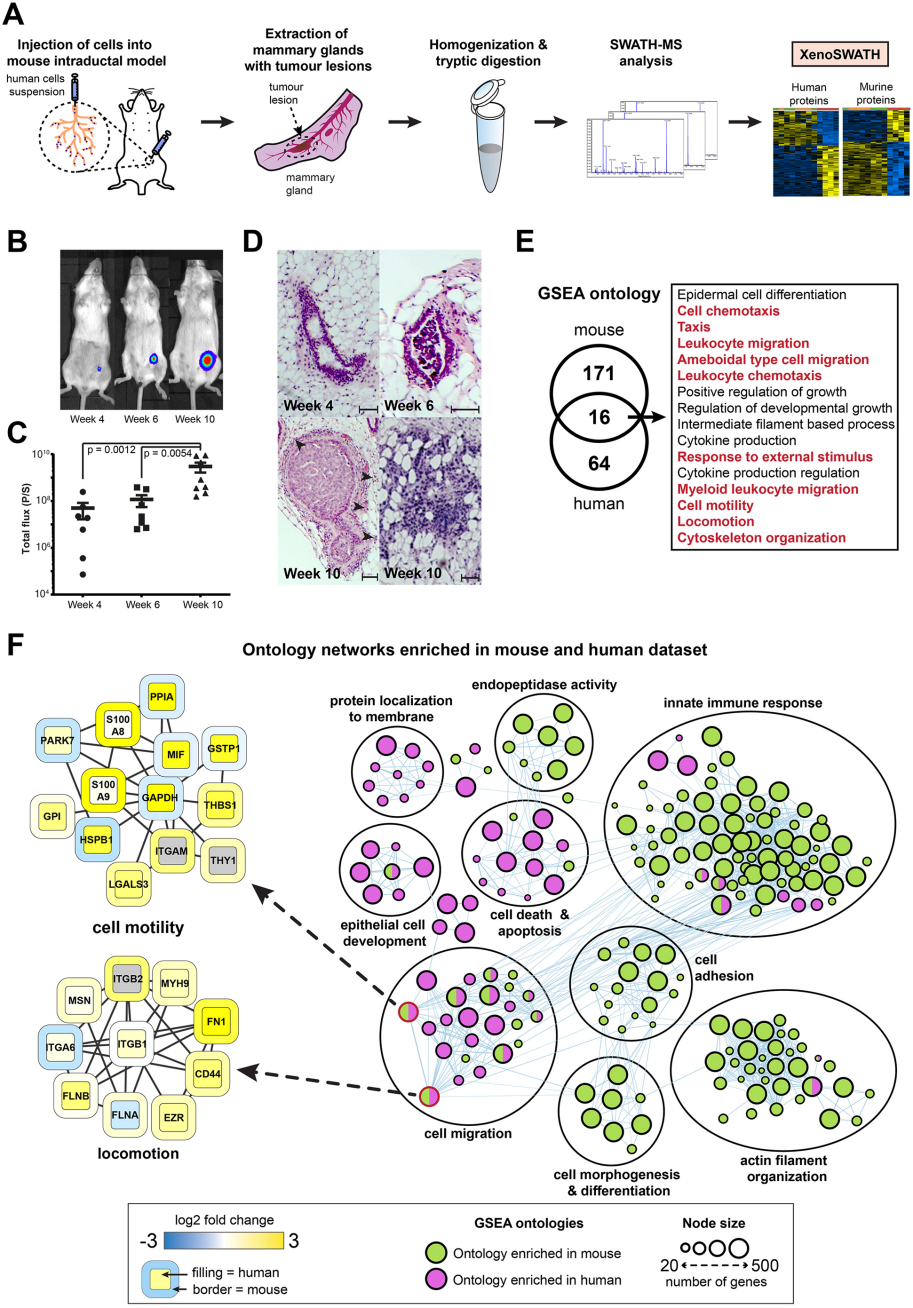
**Quantitative proteomic profiling of ductal carcinoma *in situ* (DCIS) progression to invasive breast cancer in the mouse intraductal (MIND) xenograft model.** (A) Experimental workflow of SWATH-MS analysis of tumour xenografts. MCF10DCIS.com-Luc cells were injected orthotopically into mouse mammary gland ducts through the nipple. Whole mammary glands with tumours were removed at 4, 6 and 10 weeks (w) post-injection and subjected to sample preparation prior to SWATH-MS data acquisition and analysis by the XenoSWATH pipeline. (B) Representative bioluminescence images as measured by IVIS at 4w, 6w and 10w post-injection. (C) Total bioluminescence flux reflecting tumour size in MIND model at 4w, 6w and 10w post-injection (*n*=7 for 4w and 6w samples, *n*=8 for 10w samples). *P*-value represents statistical significance of the difference between the sample groups calculated by two-tailed Mann–Whitney test. Each dot represents one biological replicate; error bars indicate mean±s.e.m. P/S, photons/s. (D) H&E images showing initial DCIS lesion formation in the MIND model at 4w, which progresses to form an invasive tumour at 10w post-injection. Tumour microinvasion in lesion 10w post-injection is indicated by arrowheads. Scale bars: 100 μm. (E) Venn diagram showing the overlap of enriched ontologies for proteins that are significantly upregulated in the 10w specimens (*n*=4) versus the 4w (*n*=4) and 6w (*n*=4) specimens as identified by gene set enrichment analysis (GSEA) with FDR-adjusted *P*-value <0.1. Ontologies associated with cell motility, migration and cytoskeleton organization are highlighted in red. (F) Network depicting ontologies enriched in human (purple) and mouse (green) datasets (10w versus 4w and 6w) and their overlap as identified by GSEA. Detailed protein networks for two selected ontologies (cell motility and locomotion) are shown. In these networks, the protein node border colour represents the mouse dataset while the protein node fill colour represents the human dataset. Only proteins with expression Log2 fold change >0.58 in either the mouse or human dataset are displayed. Proteins that are not detected are represented by grey.

We applied the XenoSWATH deconvolution pipeline to quantify the species-specific proteomic alterations associated with DCIS progression to IBC in an orthotopic mouse intraductal breast DCIS (MIND) xenograft model. The MIND model is based on the injection of human breast DCIS cells such as the MCF10DCIS.com cell line into the mouse mammary duct (Fig. 4A) (Behbod et al., 2009; Sflomos et al., 2016; Miller et al., 2000). Compared to the other breast cancer xenograft models, such as subcutaneous and mammary fat pad injection, the MIND model has been shown to better recapitulate the mammary gland microenvironment (Sflomos et al., 2016), a key regulator of breast cancer progression (Hu et al., 2008; Nelson et al., 2018), and is therefore a more clinically relevant model for this disease. In our experiments, MCF10DCIS.com-Luc cells were injected intraductally into the mammary glands of mice, where they form tumours that faithfully model the process of DCIS progression over the course of 10 weeks (Fig. 4B-D) (Behbod et al., 2009). Tumours at 4 weeks post-injection (referenced as 4w, number of biological replicates *n*=7) mimic non-invasive DCIS lesions, while after 6 weeks of growth (6w, *n*=7), tumours start microinvading into the surrounding tissue and finally progress to full IBC at 10 weeks (10w, *n*=8) post-injection (Fig. 4D). There is a significant increase in size of the 10w lesions compared to the 4w (*P*=0.0012) and 6w (*P*=0.0059) lesions but no significant difference in tumour size between 4w and 6w lesions (*P*=0.317) (Fig. 4B,C); most lesions at 6w remain within the duct and only a few cells are microinvading out of the ducts.

Whole mammary glands with tumour (*n*=4) were collected after 4w, 6w and 10w post-injection and subjected to SWATH-MS analysis (Fig. 4A). When processed through the XenoSWATH pipeline (Fig. 3), we successfully quantified 2086 murine and 1177 human proteins in all samples across the three time-points (Tables S1 and S2). Our analysis showed no significant differences in protein expression levels in both human and murine datasets between the 4w and 6w specimens, indicating that the non-invasive DCIS lesions (4w) and tumours with microinvasion (6w) harbour similar proteomic profiles at this level of resolution. Comparing the 4w and 6w data with the 10w lesions led to the identification of 327 murine and 247 human proteins that showed significant changes in protein expression (Tables S3 and S4).

Gene set enrichment analysis (GSEA) was performed to identify functional ontologies that were enriched in the murine and human proteomic datasets upon DCIS progression to IBC (Fig. 4E; Table S5). This analysis showed a number of ontology networks that were almost exclusively enriched in either the human tumour cells (cell death and apoptosis, protein localization to membrane and epithelial cell development) or the mouse stroma (cell adhesion, actin filament organization and innate immune response) (Fig. 4F). Notably, we identified 16 overlapping ontologies that were upregulated in both species, the majority of which were associated with increased cell migration (Fig. 4E,F). An assessment of the upregulated proteins (Log2 fold change >0.58) in a subset of these overlapping ontologies revealed a complex regulation of protein networks in both the human and mouse compartments. For instance, within the networks regulating cell motility and locomotion (Fig. 4F), specific proteins were upregulated in the tumour cells only [e.g. macrophage inhibitory factor (MIF) and peptidylprolyl isomerase A (PP1A)], host cells only [e.g. S100a8 and S100a9] or in both compartments [e.g. galectin-3 (LGALS3), fibronectin (FN1), ezrin (EZR), CD44 antigen (CD44)]. Employing the deconvolution pipeline, our analyses demonstrate, for the first time, that the temporal upregulation of cell migration pathways consistent with DCIS to IBC progression is not restricted only to the tumour cells but is found to also operate in the host tumour microenvironment. This proof-of-principle experiment demonstrates the utility of our XenoSWATH deconvolution pipeline for the comprehensive mapping of temporal alterations in the tumour versus host proteome in tumour xenograft models and its ability to capture relevant tumour biology for subsequent investigation.

## DISCUSSION

In this study, we have built the first comprehensive mouse reference spectral library (MouseRefSWATH) for SWATH-MS applications. The MouseRefSWATH library is generated from proteomic datasets acquired across a wide range of murine tissue samples, primary cells and cell lines, which facilitates versatile use in proteomic profiling of various sample types. We demonstrate its utility in two publicly available SWATH-MS datasets, where the use of MouseRefSWATH identified and accurately quantified >80% of the proteins compared to study-specific libraries, with a Pearson's correlation coefficient for protein quantification of at least 0.78. In both datasets, the MouseRefSWATH library further identified new proteins, which would have otherwise not been possible with study-specific libraries. Moving forward, researchers who seek to undertake a murine SWATH-MS experiment need only to download and use the publicly available MouseRefSWATH library, thereby dispensing with the requirement to generate study-specific libraries, ultimately saving time and costs.

We have also extended the use of the MouseRefSWATH library and developed a novel analysis pipeline called XenoSWATH that enables deconvolution of murine and human proteins from ‘bulk tumour’ xenograft proteomic measurements through the identification of species-discriminating proteotypic peptides. The lack of tools to perform a deep analysis of tumour (human) and host (mouse) molecular alterations *in situ* has limited our ability to study the role of the tumour microenvironment in driving tumour progression. *In silico* approaches have been developed to deconvolute mouse and human reads in next-generation DNA and RNA sequencing data derived from tumour xenografts, but such tools are limited in proteomic data analysis (Kluin et al., 2018). Prior studies using DDA-MS in human glioma and breast cancer xenografts have identified species-specific proteins in the range of 1000-2000 mouse or human proteins (Rajcevic et al., 2009; Wang et al., 2017; Wildburger et al., 2015). However, owing to the stochastic nature of this MS method, there is poor reproducibility in protein identification across independent experiments (∼50%) in some of these previous studies (Wang et al., 2017). The only published application of SWATH-MS in a xenograft model thus far has focused on measuring the murine stromal component by pre-enrichment of mouse cells with immunoaffinity chromatography while completely omitting the human tumour cells (Kalita-de Croft et al., 2019). XenoSWATH confers the ability to distinguish and reproducibly map proteomic alterations in both the human and mouse compartments with comparable protein identification numbers as DDA-MS and provides a new approach to investigating tumour cell-microenvironmental interactions in cancer initiation, progression and therapy response. With the recent development of human tumour xenograft models in immunodeficient zebrafish (Yan et al., 2019), we anticipate that XenoSWATH can be readily extended to the study of host and tumour cell responses in these models by similarly utilizing the zebrafish reference spectral library available in SWATHAtlas (Blattmann et al., 2019).

As proof of principle, we undertook a quantitative proteomic analysis in the MIND model and provide the first characterization of host stroma and tumour cell proteomic alterations that occur during DCIS to IBC progression. Notably, unlike a previous DDA-MS study of species-specific proteomic analysis in subcutaneous breast cancer xenografts (Wang et al., 2017), our study was undertaken in an orthotopic intraductal model, which better recapitulates the mammary gland microenvironment. Our analysis reveals complex network alterations in both compartments, and while the human tumour cells show an expected upregulation of migration pathways during progression to invasive disease, we make the unanticipated discovery that the mouse stroma also undergoes extensive remodelling with an enrichment of cell migration and motility networks upon DCIS progression. In the mouse dataset, the highest observed increase in migration-associated protein expression levels between early (4w and 6w) versus late (10w) time points were in the S100a8 and S100a9 proteins (Table S4). These two proteins together form the calprotectin complex, and stromal cells secreting calprotectin have been previously observed in the microenvironment of pancreatic cancer (Nedjadi et al., 2018). Additionally, S100a8 has been reported to increase the migration and proliferation of colorectal and pancreatic cancer cells *in vitro* (Nedjadi et al., 2018) and has also been associated with metastasis formation in breast cancer (Zhong et al., 2018). MIF was found to be exclusively upregulated in the human MCF10DCIS.com cells at 10w post-injection but not in the mouse stromal cells (Table S3). Increased levels of MIF have been reported in many cancer types including pancreatic cancer (Tan et al., 2014), hepatocellular cancer (Huang et al., 2014) or head and neck cancer (Kindt et al., 2013). Experiments with the murine breast cancer cell line 4T1 have shown that overexpression of MIF promotes tumour metastasis (Simpson et al., 2012) and protects cancer cells from immunogenic cell death (Balogh et al., 2018). Finally, we determined that LGALS3, CD44, FN1 and EZR were upregulated in both tumour and host stromal datasets. These proteins have been extensively shown in published literature to regulate cytoskeleton remodelling, epithelial-mesenchymal transition and increased cell motility (Pucci-Minafra et al., 2017; Mani et al., 2008; Bruce et al., 2007), all of which are important in breast cancer progression to invasive disease. These examples highlight the utility of this deconvolution pipeline in dissecting the individual roles of the tumour cells and the associated stroma in cancer biology. This dataset serves as a rich resource of tumour and microenvironmental proteins for future functional investigation, candidate biomarkers for stratifying DCIS patients with increased risk of progressing to IBC, as well as targets for drug discovery for delaying DCIS progression.

There are several limitations to the XenoSWATH pipeline. Because the method focuses on quantifying peptides that are both proteotypic and species discriminating, a significant number of proteins with high sequence similarity between mouse and human are filtered out and lost during the process, resulting in the quantification of a reduced subset of the proteome. Despite this limitation, we are still able to readily identify key pathways that are operating in both the human and mouse compartment. Given that the stroma is composed of a number of different cell types including fibroblasts, endothelial cells and immune cells, as with other population-level based measurement methodologies, the XenoSWATH pipeline is unable to resolve the individual contribution of specific stromal cell types to the aggregate proteomic data. Extending *in silico* transcriptomic deconvolution strategies that are currently being used to estimate stromal cell types to proteomic data may provide better resolution and address this shortcoming in the future (Sturm et al., 2019).

Collectively, our study has generated the MouseRefSWATH comprehensive mouse reference spectral library as a standardized community resource for use in future mouse SWATH-MS studies, which will not only remove the need for the generation of study-specific libraries but will also improve inter-laboratory reproducibility. We further present a new XenoSWATH analysis pipeline for species-specific deconvolution of xenograft proteomic data, which opens new possibilities for the in-depth and reproducible assessment of tumour-host interactions in murine xenograft models. Moving forward, we anticipate that these tools will have broad applications in addressing key biological research questions involving this widely used model organism.

## MATERIALS AND METHODS

### Murine organs, primary cells and cell lines

Isolation of T cells was carried out under Swiss animal experiment regulations. All other animal work was carried out under UK Home Office project and personal licences following local ethical approval from the Institutional Animal Ethics Committee Review Board and in accordance with local and national guidelines. Mammary gland (*n*=2), liver (*n*=1) and lung (*n*=2) organs were dissected from two 14-week-old to 18-week-old virgin female SCID-beige mice. Brain (*n*=2), heart (*n*=2) and kidney (*n*=1) organs were dissected from two 6-month-old female NCR nude mice. Axillary lymph nodes (*n*=6) and bronchial lymph nodes (*n*=6) were dissected from three 2- to 4-month-old C57BL/6N male and female mice. All tissue specimens were briefly washed in cold phosphate-buffered saline (PBS) to remove excess blood and immediately snap frozen in liquid nitrogen and stored at −80°C.

Primary CD8^+^ effector T cells were isolated from splenocytes of OT-I mice and cultured in Roswell Park Memorial Institute (RPMI) medium with 10% foetal bovine serum (FBS) (Gibco), 1% penicillin-streptomycin and 0.1% β-mercaptoethanol. In order to activate OT-I CD8^+^ T cells, OT-I splenocytes were treated with 1 μg/ml OVA257-264 peptides in the presence of 10 ng/ml IL-2 for 3 days, while the non-stimulated resting CD8^+^ T cells were collected after culturing with IL-2 alone. Immortalized normal (NF1) and cancer-associated (CAF1) fibroblasts (Calvo et al., 2013) were cultured in Dulbecco's modified Eagle medium (DMEM) supplemented with 10% FBS, 1× GlutaMAX (Gibco), 0.5% penicillin-streptomycin and 1× insulin-transferrin-selenium A (ITS-A) (Gibco). NIH-3T3 cells were cultured in DMEM supplemented with 10% FBS/100 units/ml penicillin/100 mg/ml streptomycin. C2C12 and 4T1 cells were cultured in RPMI supplemented with 10% FBS/100 units/ml penicillin/100 mg/ml streptomycin. Ba/F3 cells were cultured in the same media as 4T1 cells with the addition of 5 ng/ml IL-3. All cells were cultured in 95% air/5% CO_2_ atmosphere at 37°C. All cell lines were obtained from American Type Culture Collection (ATCC) and evaluated for mycoplasma contamination prior to being used for experiments.

### Tissue and cell sample processing

Tissue samples were cut into small pieces and placed into precooled tubes. All organs were processed as individual biological replicates with the exception of lymph nodes; six lymph nodes of the same type were pooled to generate sufficient material for MS analysis. High-salt homogenization buffer consisting of 50 mM Tris-HCl (pH 7.4), 0.25% 3-[(3-cholamidopropyl)dimethylammonio]-1-propanesulfonate hydrate (CHAPS, Sigma-Aldrich), 25 mM EDTA, 3 M NaCl (Sigma-Aldrich) and 10 KIU/ml aprotinin was added to 4 ml/g of tissue, and samples were homogenized by 2×30 s pulses on ice with a LabGEN125 (Cole-Parmer) homogenizer. Homogenized samples were rotated for 20 min at 4°C. Proteins in homogenate were acetone precipitated by mixing with four volumes of ice-cold acetone, vortexed and incubated for 2 h at −20°C. Samples were spun for 15 min at 15,000 ***g*** at 4°C, the supernatant was removed and the resulting pellet was resuspended in 0.3 ml urea buffer consisting of 8 M urea (Sigma-Aldrich), 100 mM ammonium bicarbonate (Sigma-Aldrich). Protein concentration in samples was measured using the Pierce 660 nm protein assay (Thermo Fisher Scientific), as per the manufacturer's instructions, and stored at −80°C until further processing.

Cells were harvested and lysed in 8 M urea buffer and protein concentration was measured by Pierce BCA assay (Thermo Fisher Scientific) as per the manufacturer's instructions. Cell lysates were stored at −80°C until further processing.

For each sample lysate, 200 µg total protein was reduced with 20 mM dithiothreitol (Sigma-Aldrich) at 56°C for 40 min and alkylated by 30 mM iodoacetamide (Sigma-Aldrich) at room temperature for 25 min in the dark. Samples were diluted to a final concentration of 2 M urea, 100 mM ammonium bicarbonate and digested at 37°C overnight with 4 µg sequencing-grade trypsin (Promega). Digestion was stopped by acidification to pH<4 with trifluoracetic acid (Sigma-Aldrich), and resulting peptides were desalted on SepPak C18light (Waters) cartridges as per the manufacturer's instructions. Desalted peptides were dried in a SpeedVac concentrator and stored at −20°C until fractionation.

### Fractionation by SCX chromatography

Dried peptides were resuspended in 100 µl buffer SCX-A, consisting of 10 mM NH_4_COOH (Sigma-Aldrich) in 20% acetonitrile (Thermo Fisher Scientific), pH 2.7, vortexed, sonicated for 5 min and spun at 15,000 ***g*** for 1 min. The supernatant was loaded on a 2.1×100 mm polysulfoethyl A column with 5 µm, 200 Å particles (PolyLC Inc.) and eluted with buffer SCX-B (500 mM NH_4_COOH in 20% acetonitrile, pH 2.7) using a gradient of 0-10% buffer SCX-B for 2.5 min, 10-50% buffer SCX-B for 20 min, 50-100% buffer SCX-B for 7.5 min and 100% buffer SCX-B for 10 min. Twelve fractions were manually collected over 39 min, with fraction 1 collected from 0 to 12 min and fraction 12 from 32 to 39 min. The remaining ten fractions were collected at 2-min intervals between 12 min and 32 min. All SCX fractions were dried in a SpeedVac concentrator.

### Fractionation by HpH-RP chromatography

Dried peptides were resuspended in 100 µl buffer HpH-A (0.1% NH_4_OH), vortexed, sonicated for 5 min and spun at 15,000 ***g*** for 1 min. The supernatant was loaded on a 2.1×150 mm XBridge BEH C18 column with 5 µm, 130 Å particles (Waters) and eluted with buffer HpH-B (0.1% NH_4_OH in acetonitrile) using a linear gradient of 0-50% buffer HpH-B over 60 min. Fractions were automatically collected into 96-well plates every 30 s between 5 min and 50 min, and fractions were pooled into 12 fractions (columns pooled) for 3T3 cells or eight fractions (rows pooled) for 4T1, BaF3 and C2C12 cells. Pooled fractions were dried in a SpeedVac concentrator.

### DDA-MS data acquisition

All fractions were resuspended in 20 µl buffer A (2% acetonitrile, 0.1% formic acid) and peptide concentration was measured using the 280 nm NanoDrop assay. One microgram of total peptide was analysed in DDA mode on an Agilent 1260 HPLC coupled to a TripleTOF 5600+ mass spectrometer equipped with NanoSource III. Each fraction was spiked with 0.1 µl of iRT calibration mix (Biognosys, AG) and loaded onto a 0.3×5 mm ZORBAX C18 (Agilent Technologies) trap column. Peptides were separated on a 75 µm×15 cm analytical column packed with Reprosil Pur C18AQ beads, 3 µm, 120 Å (Dr. Maisch, GmbH) with a manually pulled integrated spraying tip. A linear gradient of 2-40% buffer B (98% acetonitrile, 0.1% formic acid) in 90 min and flow rate of 250 nl/min was used for peptide separation. All data were measured in positive mode. Full profile MS scans were acquired in the m/z mass range of 340-1500 with 250 ms filling time; MS/MS scans for the 20 most intense ions with charge state from 2+ to 5+ were acquired in m/z mass range of 280-1500 with 100 ms filling time. Dynamic exclusion of fragmented ions was set to 12 s.

### DDA-MS data processing and MouseRefSWATH reference spectral library generation

All acquired DDA datasets were searched by SpectroMine (ver 1.0.21621.7 Sapphire) software (Biognosys AG) against a SwissProt mouse database (downloaded on 26/10/2018) with added iRT peptide sequences. Carbamidomethylation of cysteines was set as a fixed modification, oxidation of methionine and proline, deamidation of glutamine and asparagine, and acetylation of protein N-terminus were set as variable modifications. A maximum of two missed cleavage sites was allowed during the search. False discovery threshold on peptide and protein level was set to 1% to filter search results.

To generate final reference spectral library, retention times in each run were individually calibrated using iRT calibration peptides. Runs with low result of calibration fit (R^2^<0.8) were removed. To avoid inflation of FDR during the library generation from multiple datasets, Search Archives of all datasets were combined in SpectroMine and the MouseRefSWATH reference spectral library was built using the following parameters: minimum three and maximum six transitions for each precursor, library-wide protein and peptide FDR threshold of 1%. The MouseRefSWATH library is deposited in ProteomeXchange with identifier PXD017209 and in SWATHatlas.org and PeptideAtlas.org with identifier PASS01569.

### Comparative analysis of publicly available SWATH-MS data

Publicly available datasets (PXD006382, PXD005044) were downloaded from the ProteomeXchange data repository via the ProteomeCentral portal (Vizcaíno et al., 2014) and analysed by Spectronaut (version 13.6.190905) software (Biognosys AG). Either the MouseRefSWATH library or the relevant study-specific spectral library was uploaded into Spectronaut and all samples were processed using FDR threshold of 1% on peptide and protein levels. Mitochondrial proteins were identified by matching the list of quantified proteins with the MitoCarta 2.0 database (Calvo et al., 2016). It should be noted that in the original published studies, older versions of the Spectronaut software with different settings were used by the authors, which leads to the discrepancies in the number of reported quantified proteins in our study when compared to the original published data. Detailed Spectronaut settings for this study are shown in Table S6.

### MIND model

The MIND model was utilized in our studies as previously described (Behbod et al., 2009; Oliemuller et al., 2017). Briefly, a suspension of 5×10^4^ MCF10DCIS.com-Luc cells was injected intraductally into mammary gland ducts of 6- to 10-week-old SCID-beige female mice (*n*=7-8). Luminescence of the lesions was measured by *in vivo* imaging assay (IVIS) on IVIS Illumina II (Perkin Elmer) to monitor tumour growth. Whole mammary glands with tumour were collected after 4w, 6w or 10w post-injection, washed in cold PBS and freshly frozen. For each condition, four biological replicates were further processed and analysed by SWATH-MS. Haematoxylin and Eosin (H&E) staining was performed on formalin-fixed and paraffin-embedded samples and tissue images were scanned on a Nanozoomer XR (Hamamatsu Photonics) automated slide scanner.

### MIND model sample processing and SWATH-MS data acquisition

Whole mammary glands with tumours (*n*=4 for each time point) were homogenized in high-salt homogenization buffer, proteins were precipitated with ice-cold acetone at −20°C, centrifuged and the resulting pellet was resuspended in 0.3 ml urea buffer. Then, 20 µg total protein was digested in solution by trypsin as described above, desalted on OMIX tips as per the manufacturer's instructions and dried in a SpeedVac concentrator. Dried samples were resuspended in 20 µl buffer A and analysed in SWATH-MS mode on an Agilent HPLC coupled to TripleTOF 5600+ mass spectrometer and operated by Analyst 1.5 software (SCIEX). One microgram of sample was spiked with 0.1 µl iRT peptides and loaded onto a 0.3×5 mm ZORBAX C18 (Agilent Technologies) trap column. Peptides were separated on a 75 µm×15 cm analytical column packed with Reprosil Pur C18AQ beads, 3 µm, 120 Å (Dr. Maisch, GmbH) with a manually pulled integrated spraying tip. A linear gradient of 2-40% of buffer B in 120 min and flow rate of 250 nl/min was used for peptide separation. All data were acquired in positive-ion mode in SWATH mode using cycles consisting of one 100 ms profile MS scan over the m/z mass range of 340-1500, followed by 60 SWATH fragmentation windows with a fixed width of 12 Da over the m/z range of 380-1100 and filling time of 50 ms. All SWATH-MS data were acquired in technical duplicates. The SWATH-MS data are deposited in ProteomeXchange with identifier PXD017209.

### Implementing XenoSWATH species-specific deconvolution pipeline and MIND model SWATH-MS data processing

A combined FASTA file was generated in NotePad text editor (Microsoft) from individual FASTA files containing human (20,316 protein sequences), mouse (16,997 protein sequences) and iRT peptides. The human and mouse FASTA files were downloaded from SwissProt (downloaded on 26/10/2018) and the FASTA file with iRT sequences was downloaded from the Biognosys website (https://www.biognosys.com/shop/irt-kit#SupportMaterials). All entries from the mouse and iRT FASTA files were copied and inserted into the human FASTA file, which was then saved as a new combined FASTA file. The acquired MIND model SWATH-MS data were processed in Spectronaut using the MouseRefSWATH reference spectral library, the published pan-Human reference spectral library (Rosenberger et al., 2014) and the combined FASTA file. The SWATH-MS data were first searched using the MouseRefSWATH library. Using the combined FASTA file and MouseRefSWATH library, Spectronaut selects only the mouse species discriminating proteotypic peptides from the MouseRefSWATH library for the quantification of murine proteins. The same approach was repeated with the pan-Human library for the quantification of human species discriminating proteotypic peptides. All searches were performed with 1% FDR threshold on peptide and protein level (detailed Spectronaut settings are shown in Table S6). In this manner, two separate proteomic datasets were obtained – one for the tumour component (human) and the other for the host stromal compartment (mouse). The datasets were separately quantile normalized using proBatch package (Cuklina et al., 2018) in R (www.r-project.org) and statistically analysed by two-tailed Student's *t*-test with multiple testing correction (Benjamini and Hochberg, 1995) in Perseus (version 1.5.6.0, https://maxquant.net/perseus/) (Tyanova et al., 2016). Results were considered statistically significant if the FDR-adjusted *P*-value was <0.05. Analysis of ontologies enriched in 10w versus 4w and 6w was performed using a GSEA desktop application (https://www.gsea-msigdb.org/gsea/index.jsp) against human or mouse MSigDb of biological processes (Subramanian et al., 2005; Mootha et al., 2003). The FDR threshold for multiple testing correction was set to 0.1 using genotype permutations due to a low number of biological replicates. Protein networks were visualized in Cytoscape (version 3.7.1, www.cytoscape.org) (Shannon et al., 2003) and clustered by the MCL clustering algorithm in clusterMaker2 Cytoscape plugin (Morris et al., 2011). For the generation of protein networks in Cytoscape, a fold change cut-off of at least Log2 0.58 was applied.

## Supplementary Material

Supplementary information
