# Supplementary material for: A mouse SWATH-mass spectrometry reference spectral library enables deconvolution of species-specific proteomic alterations in human tumour xenografts
Source: Dis Model Mech. 2020 Jul 14;13(7):dmm044586. doi: 10.1242/dmm.044586 (PMC7375474; doi:10.1242/dmm.044586)
Supplement: Supplementary information [file dmm-13-044586-s1.pdf]

Table S1: Full proteomic dataset of human proteins obtained from MIND model experiment as processed by XenoSWATH species-specific deconvolution pipeline. Confidence of the protein identification is reported as FDR-adjusted p-value.

Click here to Download Table S1

Table S2: Full proteomic dataset of mouse proteins obtained from MIND model experiment as processed by XenoSWATH species-specific deconvolution pipeline. Confidence of the protein identification is reported as FDR-adjusted p-value.

Click here to Download Table S2

Table S3: List of human proteins with significantly altered expression in 10w samples compared to 4w & 6w samples. Log2 fold change (Log2FC) for each protein is reported along with the FDR-adjusted p-value from the two-tailed Student's t-test.

Click here to Download Table S3

Table S4: List of mouse proteins with significantly altered expression in 10w samples compared to 4w & 6w samples. Log2 fold change (Log2FC) for each protein is reported along with the FDR-adjusted p-value from the two-tailed Student's t-test.

Click here to Download Table S4

Table S5: Overlapping ontologies enriched in both human and mouse dataset (Figure 4E). GSEA normalized enrichment scores (NES) and FDR-adjusted p-values are shown based on analysis of 10w versus 4w & 6w samples.

Click here to Download Table S5

Table S6: Detailed Spectronaut settings used for XenoSWATH analysis

Click here to Download Table S6
